# A Cognitive Reserve and Social Support-Focused Latent Class Analysis to Predict Self-Reported Confusion or Memory Loss among Middle-Aged World Trade Center Health Registry Enrollees

**DOI:** 10.3390/ijerph16081401

**Published:** 2019-04-18

**Authors:** Kacie Seil, Shengchao Yu, Howard Alper

**Affiliations:** New York City Department of Health and Mental Hygiene, World Trade Center Health Registry, 125 Worth Street, New York, NY 10013, USA; syu@health.nyc.gov (S.Y.); halper@health.nyc.gov (H.A.)

**Keywords:** cognitive reserve, cognitive decline, latent class analysis, disaster epidemiology, PTSD

## Abstract

The World Trade Center Health Registry includes 9/11 survivors who have been surveyed about their health conditions over time. The prevalence of posttraumatic stress disorder (PTSD) remains high among the cohort and is a risk factor for cognitive impairment or dementia. We thus sought to examine the degree to which confusion or memory loss (CML)—potential symptoms of cognitive decline—are occurring among enrollees aged 35–64 years. Cognitive reserve theory suggests that higher levels of education and engaging in cognitively challenging activities can create stronger neural connections, offering protection against cognitive decline. We hypothesized that enrollees with less cognitive reserve would be more likely to report CML. In this study, we: (1) estimated the incidence of CML in our study sample; (2) identified indicators of cognitive reserve (e.g., indicators of educational attainment, social support); and (3) determined whether CML is associated with cognitive reserve level, stratified by PSTD status. First, we described demographics of the study sample (*n* = 14,574) and probable PTSD status, also stratifying by CML. Next, we conducted a latent class analysis on two groups: those with probable PTSD and those without probable PTSD, creating classes with varying cognitive reserve levels. Finally, using adjusted log binomial models, we predicted risk of CML based on cognitive reserve level. The probable PTSD group (*n* = 1213) and not probable PTSD group (*n* = 13,252) each had four latent classes: low, medium-low, medium-high, and high cognitive reserve. In the probable PTSD model, compared to the high cognitive reserve class, those with medium-low cognitive reserve were 35% more likely to report CML (relative risk (RR) = 1.4, 95% confidence interval (CI): 1.1, 1.7). Among the not probable PTSD group, those with low and medium levels of cognitive reserve were significantly more likely to report CML (RR = 1.8 and 1.4, respectively). Overall, those with less cognitive reserve were more likely to report CML regardless of PTSD status.

## 1. Introduction

The terrorist attacks on the World Trade Center (WTC) in New York City (NYC) on 11 September 2001 resulted in thousands of deaths and non-fatal injuries. Survivors of the attacks also witnessed traumatic events and were exposed to dust and debris related to the towers’ collapse, the health effects of which include posttraumatic stress disorder (PTSD), asthma, cancer, and other conditions [1,2,3]. Seventeen years after the event, survivors continue to exhibit effects. As the cohort ages, the prevalence of chronic conditions associated with older age will likely increase. Mild to severe forms of cognitive decline affect a large number of Americans, particularly those over the age of 65 years, though they can affect younger individuals as well [4,5]. 

Co-morbid health conditions can put one at greater risk for cognitive decline; PTSD is associated with greater likelihood of developing cognitive impairment or various forms of dementia [6,7,8,9,10,11,12]. It is possible that 9/11-related exposures and resulting health conditions may put enrollees at greater risk for cognitive decline than the general population [11,13]. Research has shown elevated levels of PTSD among the WTC Health Registry (WTCHR) cohort in the years following the disaster [1,14]. Research by Clouston et al. (2017) examined WTC responders from the WTC Health Program and found that 14.8% of responders had cognitive dysfunction identified via a battery of tests. This proportion was larger than expected compared to normative data from age-matched healthy adults. Cognitive dysfunction was also associated with PTSD symptom severity and working at the 9/11 site for more than five weeks [11]. 

People who are less socially isolated (i.e., engaging in more social activities and having larger social networks) are less likely to have cognitive problems later in their lives [15,16]. Research has also shown that increases in “out of home” activities and walking duration can be protective against cognitive impairment as well [17]. Similarly, social interaction and cognitive control skills specific to certain kinds of occupations appear to be protective against neurodegeneration [18,19,20,21,22]. Engaging in cognitively stimulating activities is also positively associated with cognition [22,23]. Research shows that greater levels of cognitive reserve may help to stave off the symptoms of early forms of cognitive decline, leading to more time spent in good health [24,25]. Cognitive reserve theory suggests that higher levels of education, as well as engaging in cognitively challenging activities, can create stronger neural connections, which may offer protection against cognitive decline symptoms [5,20,22,25,26,27,28]. The more cognitive reserve a person has, the more plastic and adaptable their brain is; a brain with more plasticity can handle damage more effectively before showing clinical symptoms [25,29,30,31].

Questions about confusion and memory loss were included on the two most recent WTCHR major surveys, allowing researchers to better understand the degree to which early signs of cognitive decline may be occurring among this population. Rather than assessing the direct link between 9/11-related health conditions, such as PTSD and cognitive decline, this study focused on identifying certain individual-level factors that may be protective against confusion or memory loss among WTCHR enrollees. In this study, self-reported confusion or memory loss is a surrogate measure for potential cognitive issues. It is not intended to be a substitute for cognitive testing or a clinical diagnosis. 

The goals of this study were to: (1) estimate the degree of self-reported confusion or memory loss among those exposed to the WTC attacks; (2) identify indicators of cognitive reserve (with a particular focus on social support) that can be created from survey variables (i.e., indicators of educational attainment, marital status, employment status, social support, social integration, and physical activity); and (3) determine whether confusion or memory loss is associated with differing levels of cognitive reserve. We hypothesized that groups with less cognitive reserve would be more likely to report confusion or memory loss. 

## 2. Materials and Methods 

### 2.1. Study Population and Sample

The WTCHR maintains a longitudinal cohort of over 71,000 enrollees who were exposed to the terrorist attacks on 11 September 2001. Enrollees in the registry have been surveyed about their exposures to the disaster as well as their short- and long-term health effects in four major surveys: wave 1 (2003–2004), wave 2 (2006–2007), wave 3 (2011–2012), and wave 4 (2015–2016). Health effects will continue to be monitored for years to come. Enrollees include rescue/recovery workers, residents, area workers, passers-by, and students and staff from local schools. The WTCHR was approved by the institutional review boards of the Centers for Disease Control and Prevention (CDC) (3793) and the NYC Department of Health and Mental Hygiene (02-058).

**Figure 1:**
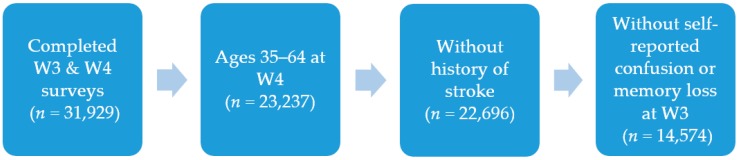
Study sample inclusion criteria.

The study sample consisted of enrollees in the WTCHR who met certain criteria (see Figure 1). Individuals in the study had to have completed the wave 3 and wave 4 surveys, both of which contained the self-reported confusion or memory loss questions. Because we were interested in the earlier/milder forms of cognitive decline, we limited our sample to enrollees who were between the ages of 35 and 64 years at the time of the wave 4 survey. We excluded those with a history of stroke [11]. Finally, individuals who reported confusion or memory loss at wave 3 were excluded from the study sample, as we wanted the majority of the predictor variables used in the analysis to precede the outcome of interest, which was confusion or memory loss at wave 4. This also allowed for a better approximation of cognitive decline, as the outcome was measuring a change in self-reported confusion or memory loss between wave 3 and wave 4. The total study sample included 14,574 enrollees. 

### 2.2. Study Variables

The outcome of interest was confusion or memory loss measured by the wave 4 question as follows: “During the last 12 months, have you experienced confusion or memory loss, other than occasionally forgetting the name of someone you recently met?” Cognitive reserve was measured by seven dichotomized indicators: educational attainment, marital status, employment status, number of close friends, communication with friends in last 30 days, people who understand your problems, and general physical activity. PTSD status at wave 3 was classified using the total score from the Post-Traumatic Stress Disorder Checklist (PCL) scale [1,32]. The scale includes measures of re-experiencing, avoidance, and arousal symptoms, and a score of 44 or greater indicates probable PTSD. Demographic factors and other covariates used in this study included gender, age group, race/ethnicity, history of depression, history of anxiety disorder, history of drug or alcohol use problems, and smoking status.

### 2.3. Data Analysis

First, we described the study sample by basic demographics, probable PTSD status, and the seven indicators that measured cognitive reserve. Then, we performed a latent class analysis and identified a latent class membership, or cognitive reserve level in this case, for each individual in the study sample based on the seven indicators mentioned above. Latent class analysis is a process through which distinct classes are created using a set of chosen variables wherein each record gets assigned to one latent class [33]. We used SAS PROC LCA [34], a statistical procedure for latent class analysis, and reviewed the outputted statistics to assess model fit, comparing the fit for 2, 3, 4, and 5 classes [33,35]. Determining that four classes provided the optimal fit, we then grouped the classes by probable PTSD status, as we knew it was likely to be a risk factor for confusion or memory loss. Latent class composition differed by probable PTSD status, so we conducted two separate latent class analyses: one for those with probable PTSD and one for those without probable PTSD. Those analyses resulted in four latent classes for each group. Next, we predicted the risk of confusion or memory loss in wave 4 by latent class membership (i.e., cognitive reserve level) using log binomial modeling. We chose this method because it produces an unbiased estimate of the relative risk whether the disease is rare or common, whereas logistic regression produces odds ratios, which approximate the relative risk for only rare diseases [36]. We ran adjusted models on the probable PTSD group and not probable PTSD group, adjusting for most of the covariates described above. We did not include age group in the models because we did not expect it to be a major predictor of the outcome, largely due to our study sample being limited to middle-aged enrollees. All analyses were completed with SAS 9.4 software.

## 3. Results

Self-reported confusion or memory loss was common among the study sample, despite the age group being limited to middle-aged individuals—those between the ages of 35 and 64 years (see Table 1). A total of 3262 out of 14,574 (22%) enrollees in the study sample reported confusion or memory loss at wave 4. About 8% of enrollees had probable PTSD at wave 3, and the majority of the study sample was male (62%). There were twice as many enrollees between the ages of 55 and 64 years in this study compared to those between the ages of 35 and 44 years (43% vs. 19%, respectively). In the total study sample, 60% of enrollees had at least a bachelor’s degree. Most enrollees were married or living with a partner in wave 3: 72%. More than four out of five enrollees (84%) were currently employed at the time of the wave 3 survey. Overall level of social integration was high among the study sample; nearly all respondents reported having at least three close friends and having visited, talked, or emailed with friends at least twice in the past 30 days (87% and 94%, respectively). Two-thirds of enrollees reported high levels of social support—specifically, that someone was available to understand their problems most or all of the time. Over three-quarters of the study sample reported being very or somewhat physically active in general. 

When stratifying these factors by confusion or memory loss status, there were some proportional differences. The proportion of probable PTSD was much greater among those with confusion or memory loss compared to those without confusion or memory loss (17% vs. 6%, respectively). Among those with confusion or memory loss, 50% had a bachelor’s degree or higher level of education; 62% of those without confusion or memory loss had at least a bachelor’s degree. Higher levels of social support were reported by those without confusion or memory loss: 71% of them had people available to understand their problems compared to 58% of those who reported having confusion or memory loss. Similarly, a larger proportion of those without confusion or memory loss reported being very or somewhat physically active compared to those with confusion or memory loss (81% vs. 70%, respectively).

The initial latent class analysis on the total study sample indicated that class membership differed significantly based on probable PTSD status at wave 3 (results not shown). However, for both the probable PTSD group and the not probable PTSD group, four classes were determined to be optimal based on the model fit statistics from the latent class analysis. The four classes were assigned as follows in both groups: low cognitive reserve, medium-low cognitive reserve, medium-high cognitive reserve, and high cognitive reserve (referent). 

Among those with probable PTSD, class 1, the low cognitive reserve class, constituted about 15% of the group (see Table 2). This group had low levels of social integration and social support (e.g., only 8% of class 1 members reported that someone was available to understand their problems most or all of the time); members were also characterized by overall lower levels of educational attainment, being less likely to be married, having medium levels of current employment, and having lower levels of physical activity. Notable differences between class 2 (8%, medium-low cognitive reserve) and class 3 (37%, medium-high cognitive reserve) are that class 2 had low levels of educational attainment, and class 3 had low levels of social support. Class 4 (40%, high cognitive reserve) was the referent group: having medium levels of educational attainment, being very likely to be married, reporting high levels of social integration and support, having medium levels of current employment, and reporting medium levels of physical activity.

Similar results were presented in Table 2 for those who did not have probable PTSD. Class 4 (high cognitive reserve) made up the majority of the not probable PTSD group: 61%. This group was the referent due to the high proportion of the cognitive reserve indicators therein:, with 73% having at least a bachelor’s degree, 79% married or living with a partner, 90% being currently employed, 97% having three or more close friends, 100% who visited/talked/emailed with friends at least twice in last 30 days, 93% having someone available to understand your problems most or all of the time, and 86% being very or somewhat physically active in general. 

Using the latent class groups as predictors for confusion or memory loss at wave 4, we ran separate log binomial models for those with probable PTSD and those without probable PTSD, controlling for relevant covariates (see Table 3). The medium-low cognitive reserve group was significantly more likely to report confusion or memory loss compared to the high cognitive reserve group among the probable PTSD group (relative risk (RR) = 1.35, 95% confidence interval (CI): 1.08, 1.69). Significant covariates in the probable PTSD model included female gender (RR = 0.84, 95% CI: 0.72, 0.97) and history of drug or alcohol use problems (RR = 1.26, 95% CI: 1.03, 1.53). Other covariates approached statistical significance, as did the relative risks for the low cognitive reserve and medium-high cognitive reserve groups. Among the non-probable PTSD group, however, every cognitive reserve group was at significantly greater risk of confusion or memory loss compared to the referent. The low cognitive reserve group was 81% more likely than the high cognitive reserve group to report confusion or memory loss at wave 4. The medium-low and medium-high groups were approximately 40% more likely to report confusion or memory loss compared to the referent. Significant covariates in the not probable PTSD group model included black non-Hispanic race (RR = 1.16, 95% CI: 1.02, 1.31), other race (RR = 1.19, 95% CI: 1.04, 1.35), history of depression (RR = 1.26, 95% CI: 1.14, 1.39), history of anxiety disorder (RR = 1.13, 95% CI: 1.00, 1.27), history of drug or alcohol use problems (RR = 1.40, 95% CI: 1.20, 1.65), being a former smoker (RR = 1.20, 95% CI: 1.11, 1.29), and being a current smoker (RR = 1.18, 95% CI: 1.05, 1.33).

## 4. Discussion

Our hypothesis was that enrollees with greater levels of cognitive reserve would be less likely to report confusion or memory loss, and our results support this. The relationship between cognitive reserve and confusion or memory loss was similar regardless of PTSD status. For both the probable PTSD and not probable PTSD groups, we found that groups with greater cognitive reserve were less likely to report confusion or memory loss, although the association was more significant and stronger for the group without probable PTSD. This finding suggested that for those who did not have probable PTSD, cognitive reserve factors may play an even more important role in modifying or delaying cognitive decline processes among 9/11 survivors. 

Cognitive reserve indicators in this study included educational attainment, social integration, social support, marital status, current employment status, and physical activity. For simplicity, the four classes for both groups (probable PTSD and not probable PTSD) were assigned equivalent names (i.e., low, medium-low, medium-high, or high cognitive reserve) despite their composition differing somewhat between the two groups. Generally, the medium-high, medium-low, and low cognitive reserve groups were at greater risk of having confusion or memory loss at wave 4 compared to the high cognitive reserve groups. There was an interesting discrepancy among the probable PTSD group in that the medium-low cognitive reserve group actually had the greatest relative risk, suggesting that it was the true low cognitive reserve group. In looking at the composition of the medium-low group, we posit that the very low probability of individuals having a bachelor’s degree or higher is the factor driving the increased risk for confusion or memory loss.

Theories on cognitive reserve are rooted in research showing that neural connections can change throughout an individual’s life [37]—often described as brain plasticity [25,37]. Individuals with greater cognitive reserve are able to process cognitive information more efficiently or use different strategies to reason through issues [27], even using additional brain regions related to memory task performance [38,39,40]. Even later in life, positive behaviors that increase one’s cognitive reserve can have an effect. For instance, cognitive training that promotes memory control and strategies for real-life memory challenges has been shown to be beneficial among older adults [41]. Although educational attainment is often the simplest and most direct proxy for cognitive reserve, our results show that other modifiable factors may be instrumental in protecting against confusion or memory loss as well. People who are active (physically and mentally) and those who engage or feel supported by others have better cognitive outcomes than those who are most isolated. Being socially engaged with others can be cognitively demanding, helping to boost one’s level of cognitive reserve [15]. 

A healthy lifestyle and better cognitive functioning are protective against countless other negative health outcomes as well [42,43,44]. Maximizing time lived in good health has a positive impact on the individual and societal levels. Greater life expectancy typically involves more years lived with cognitive impairment. However, greater educational attainment (and presumably, greater cognitive reserve) has proven to be associated with increased life expectancy and fewer years of cognitive impairment [25].

Gender, race/ethnicity, history of depression, history of drug or alcohol use problems, and smoking status were also associated with the outcome in our models. Existing literature has suggested that memory issues are correlated with anxiety and depression [45]. The nature of psychiatric comorbidities is complex, and multiple studies have concluded that risk of cognitive decline is greater among those with comorbid PTSD and depression [8,46], though depression on its own may not increase risk [46]. In fact, cognitive reserve may exert more influence than depression; Lee et al. determined that among those with greater levels of educational attainment, better memory function was seen regardless of depressive mood [28]. Ambient air pollutants or fine particulate matter may be associated with declines in domain-specific cognitive functions or development of mild cognitive impairment [13,47], but the composition of the dust cloud resulting from the attacks of 11 September 2001 differed from these exposures in composition and duration [48]. For these reasons, we did not include this type of exposure as a covariate.

Limitations of this study include limited generalizability to the broader NYC or United States population; enrollees in the WTCHR are a trauma-exposed population with a high prevalence of PTSD. Generalizability may be further limited because we excluded people with confusion or memory loss at wave 3, thereby lowering the proportions of confusion or memory loss at wave 4 as well as proportions of PTSD at wave 3; our study sample was healthier than the source population from which it was derived. Another limitation arises because we did not perform cognitive testing on enrollees, so we cannot easily compare our results to research that used this approach. A third limitation is that the questions from the WTCHR survey are very similar to those from the Behavioral Risk Factor Surveillance System [4,49], but they are not identical, so we cannot compare our numbers to those from the general population. Another limitation is that the validity of self-reported cognitive measures can be questionable, particularly for individuals who do have symptoms of impairment. However, those with mild cognitive impairment do not underreport their cognitive issues as much as those with dementia [50]. Also, our study sample is limited to middle-aged enrollees who are less likely to suffer from severe cognitive issues than older adults. Response bias and social desirability bias may lead to an underestimate of the outcome if individuals do not accurately report their confusion or memory loss as well. Finally, the probable PTSD group was much smaller than the not probable PTSD group (8% vs. 92% of total study sample, respectively), meaning that statistical power could have been limited for that group.

Strengths of this study include the fact that we were able to describe change in confusion or memory loss between wave 3 and wave 4, enabling us to approximate the outcome as mild cognitive decline. Also, the WTCHR cohort is a diverse cohort of approximately 71,000 people, giving us sufficient statistical power to detect relevant associations. Our results also support other research that has found that PTSD is an important risk factor for cognitive decline. Among our study sample, the incidence of confusion or memory loss among those with probable PTSD at wave 3 was 45%, while the incidence among those without probable PTSD was 20%.

## 5. Conclusions

There are elevated levels of PTSD among the WTCHR cohort [1,14]. Because PTSD is associated with cognitive impairment [6,7,8,9,10,11,12], we examined the degree to which confusion or memory loss was reported among our study sample. We were interested in how cognitive reserve levels might affect confusion or memory loss, as many of these factors are modifiable. Despite a high prevalence of PTSD among the source population, the majority of the cohort does not have PTSD; those individuals may still be at risk for cognitive decline. Our findings show that confusion and memory loss affect more than one in five enrollees in our study sample, and level of cognitive reserve affects likelihood of developing confusion or memory loss. Those with higher educational attainment, more social support, and greater levels of physical activity are less likely to report confusion or memory loss than those with less cognitive reserve overall. Three of the seven indicators used to create cognitive reserve levels in this study highlighted social support or social integration; their presence helps decrease likelihood of reporting symptoms of cognitive impairment. Results suggest that this is true for those with probable PTSD as well as those without probable PTSD, though the effects were stronger for those without probable PTSD. We believe that all members of the cohort could benefit from engaging in activities that promote cognitive reserve, resulting in lower likelihood of experiencing confusion or memory loss and improved overall health.

## Figures and Tables

**Table 1:** Description of study sample demographics and cognitive reserve indicators.

Sample Characteristics	Total Study Sample (*n* = 14,574) ^1,2^ *n* (%)	Confusion or Memory Loss at W4 (*n* = 3262, 22%) ^1,2^ *n* (%)	No Confusion or Memory Loss at W4 (*n* = 11,312, 78%) ^1,2^ *n* (%)
**Probable PTSD**			
Yes	1213 (8.3%)	550 (16.7%)	663 (5.9%)
No	13,252 (90.9%)	2675 (82.0%)	10,577 (93.5%)
**Gender**			
Male	8975 (61.6%)	2016 (61.8%)	6959 (61.5%)
Female	5599 (38.4%)	1246 (38.2%)	4353 (38.5%)
**Age group**			
35–44 years	2827 (19.4%)	570 (17.5%)	2257 (20.0%)
45–54 years	5526 (37.9%)	1277 (39.2%)	4249 (37.6%)
55–64 years	6221 (42.7%)	1415 (43.4%)	4806 (42.5%)
**Educational attainment**			
Bachelor’s degree or more	8693 (59.7%)	1666 (51.1%)	7027 (62.1%)
Less than a bachelor’s degree	5820 (39.9%)	1576 (48.3%)	4244 (37.5%)
Marital status			
Married or living with partner	10,482 (71.9%)	2269 (69.6%)	8213 (72.6%)
Divorced/separated, widowed, or never married	4033 (27.7%)	979 (30.0%)	3054 (27.0%)
**Employment status**			
Currently employed	12,193 (83.7%)	2671 (81.9%)	9522 (84.2%)
Not currently employed	2335 (16.0%)	579 (17.8%)	1756 (15.5%)
**Number of close friends**			
Have 3 or more close friends	12,719 (87.3%)	2720 (83.4%)	9999 (88.4%)
Have 0–2 close friends	1452 (10.0%)	448 (13.7%)	1004 (8.9%)
**Communicate with friends**			
Visited/talked/emailed with friends at least twice in last 30 days	13,681 (93.9%)	2950 (90.4%)	10,731 (94.9%)
Did not visit/talk/email with friends at least twice in last 30 days	729 (5.0%)	265 (8.1%)	464 (4.1%)
**People who understand your problems**			
Someone is available to understand your problems most or all of the time	9952 (68.3%)	1888 (57.9%)	8064 (71.3%)
Someone is available to understand your problems none to some of the time	4401 (30.2%)	1311 (40.2%)	3090 (27.3%)
**Physical activity**			
Very or somewhat physically active in general	11,413 (78.3%)	2287 (70.1%)	9126 (80.7%)
Not or not very physically active in general	3096 (21.2%)	951 (29.2%)	2145 (19.0%)

^1^ Column percentages may sum to <100% due to missing data. ^2^ Column percentages may sum to >100% due to rounding. PTSD: posttraumatic stress disorder; W4: wave 4.

**Table 2:** Latent class membership by probable PTSD status based on cognitive reserve indicators.

Cognitive Reserve Indicators	Probable PTSD (*n* = 1,213) ^1,2^				Not Probable PTSD (*n* = 13,252) ^1,2^			
	Class 1 (14.5%)	Class 2 (8.3%)	Class 3 (36.9%)	Class 4 (40.3%)	Class 1 (5.1%)	Class 2 (15.6%)	Class 3 (18.4%)	Class 4 (60.9%)
**Proportion in each latent class**								
Bachelor’s degree or more	0.345	0.050	0.588	0.504	0.331	0.183	0.673	0.725
Married or living with partner	0.572	0.665	0.466	0.797	0.772	0.817	0.445	0.792
Currently employed	0.747	0.080	0.768	0.809	0.778	0.697	0.850	0.895
Have three or more close friends	0.398	0.728	0.742	0.901	0.421	0.955	0.818	0.969
Visited/talked/emailed with friends at least twice in last 30 days	0.245	0.766	0.998	0.966	0.527	0.939	0.978	0.995
Someone is available to understand your problems most or all of the time	0.083	0.432	0.036	0.866	0.266	0.880	0.005	0.933
Very or somewhat physically active in general	0.491	0.255	0.662	0.730	0.627	0.724	0.725	0.859

^1^ 109 records did not have valid Post-Traumatic Stress Disorder Checklist (PCL) scores at wave 3 (W3), so the total does not sum to *n* = 14,574; ^2^ Numbers presented are probability of indicator among that class.

**Table 3:** Adjusted log binomial model to predict confusion or memory loss by probable PTSD status.

Sample Characteristics	Probable PTSD *n* = 1213		Not Probable PTSD *n* = 13,252	
	RR (95% CI)	p-Value	RR (95% CI)	p-Value
**Latent class**				
Class 1: low cognitive reserve	1.13 (0.90, 1.47)	0.292	1.81 (1.55, 2.11)	<0.0001
Class 2: medium-low cognitive reserve	1.35 (1.08, 1.69)	0.008	1.36 (1.21, 1.52)	<0.0001
Class 3: medium-high cognitive reserve	1.15 (0.98, 1.34)	0.080	1.44 (1.33, 1.56)	<0.0001
Class 4: high cognitive reserve	Referent	--	Referent	--
**Gender**				
Female	0.84 (0.72, 0.97)	0.017	0.93 (0.86, 1.00)	0.055
Male	Referent	--	Referent	--
**Race/ethnicity**				
White non-Hispanic	Referent	--	Referent	--
Black non-Hispanic	1.06 (0.84, 1.34)	0.609	1.16 (1.02, 1.31)	0.020
Hispanic	1.09 (0.90, 1.32)	0.376	1.06 (0.95, 1.20)	0.304
Other races	0.89 (0.67, 1.20)	0.451	1.19 (1.04, 1.35)	0.010
**Health conditions**				
History of depression	1.15 (0.99, 1.33)	0.074	1.26 (1.14, 1.39)	<0.0001
History of anxiety	1.01 (0.86, 1.18)	0.908	1.13 (1.00, 1.27)	0.050
History of drug or alcohol use problems	1.26 (1.03, 1.53)	0.024	1.40 (1.20, 1.65)	<0.0001
**Smoking status**				
Never smoker	Referent	--	Referent	--
Former smoker	1.11 (0.95, 1.29)	0.187	1.20 (1.11, 1.29)	<0.0001
Current smoker	1.16 (0.98, 1.38)	0.087	1.18 (1.05, 1.33)	0.005
